# Brain structural and functional correlates to defense-related inhibition of muscle sympathetic nerve activity in man

**DOI:** 10.1038/s41598-022-05910-8

**Published:** 2022-02-07

**Authors:** Bushra Riaz, John J. Eskelin, Linda C. Lundblad, B. Gunnar Wallin, Tomas Karlsson, Göran Starck, Daniel Lundqvist, Robert Oostenveld, Justin F. Schneiderman, Mikael Elam

**Affiliations:** 1 MedTech West, Sahlgrenska University Hospital, Roda straket 10B, 413 45 Gothenburg, Sweden; 2 Department of Clinical Neuroscience, Institute of Neuroscience and Physiology, Sahlgrenska Academy at University of Gothenburg, 413 45 Gothenburg, Sweden; 3 Department of Clinical Neurophysiology, Sahlgrenska University Hospital, 413 45 Gothenburg, Sweden; 4 Department of Medical Physics and Biomedical Engineering, Department of Medical Radiation Sciences, Sahlgrenska University Hospital and Institute of Clinical Sciences, Sahlgrenska Academy at University of Gothenburg, 413 45 Gothenburg, Sweden; 5 NatMEG, Department of Clinical Neuroscience, Karolinska Institutet, 171 77 Stockholm, Sweden; 6 Donders Institute for Brain, Cognition and Behaviour, Radboud University, 6500 HB Nijmegen, The Netherlands

**Keywords:** Autonomic nervous system, Neurophysiology, Sensory processing, Biomarkers, Preclinical research, Translational research, Risk factors

## Abstract

An individual’s blood pressure (BP) reactivity to stress is linked to increased risk of hypertension and cardiovascular disease. However, inter- and intra-individual BP variability makes understanding the coupling between stress, BP reactivity, and long-term outcomes challenging. Previous microneurographic studies of sympathetic signaling to muscle vasculature (i.e. muscle sympathetic nerve activity, MSNA) have established a neural predictor for an individual’s BP reactivity during short-lasting stress. Unfortunately, this method is invasive, technically demanding, and time-consuming and thus not optimal for widespread use. Potential central nervous system correlates have not been investigated. We used MagnetoEncephaloGraphy and Magnetic Resonance Imaging to search for neural correlates to sympathetic response profiles within the central autonomic network and sensorimotor (Rolandic) regions in 20 healthy young males. The main correlates include (a) Rolandic beta rebound and an anterior cingulate cortex (ACC) response elicited by sudden stimulation and (b) cortical thickness in the ACC. Our findings highlight the involvement of the ACC in reactions to stress entailing peripheral sympathetic responses to environmental stimuli. The Rolandic response furthermore indicates a surprisingly strong link between somatosensory and autonomic processes. Our results thus demonstrate the potential in using non-invasive neuroimaging-based measures of stress-related MSNA reactions, previously assessed only using invasive microneurography.

## Introduction

Cardiovascular disease is the leading cause of death worldwide and high blood pressure (BP) is the most important risk factor for global disease burden^1^. Approximately half of all cases are diagnosed with essential hypertension for which there is no unifying explanation. So far, while epidemiological research has identified many risk factors, it has been difficult to develop useful individual profiles for the management of hypertension, from prediction and early diagnosis to personalized treatment. Furthermore, clinical trials of anti-hypertensive medication have relied on large patient groups, mostly considered to be homogenous regarding their condition. Thus, understanding of specific disease mechanisms for essential hypertension, and related tools for research and clinical use, are still lacking. There is, however, evidence from large-scale longitudinal studies indicating that environmental stress is an important risk factor for essential hypertension^2,3^. Neural regulation is thus likely to be of importance.

Acute stress is often triggered by a sudden stimulus (e.g., a car honking, or an unexpected notification on a mobile) initiating a defense reaction. This transitory response often includes increases in heart rate, BP, and blood flow to skeletal muscles^4^. The muscle flow response is modulated via inhibition of ongoing bursts of efferent sympathetic vasoconstrictor nerve traffic to the skeletal muscle vascular bed (i.e. muscle sympathetic nerve activity; MSNA), and the resulting muscle vasodilation may buffer the BP increase generated by simultaneous activation of other sympathetic (e.g. renal/splanchnic and cardiac) subdivisions. Interestingly, any alerting stimulus, be it somatosensory, visual, or auditory, evokes similar defense patterns, but the associated MSNA inhibitory responses differ markedly among individuals^5–7^. Whereas all individuals respond with activation of sympathetic nerve traffic to skin^8–10^ approximately 50% of previously studied individuals show significant inhibition in MSNA (hereafter referred to as Inhibitors; previous publications from our group refer to them as Responders) whereas others either inhibit MSNA weakly or (in ~ 5% of individuals) increase MSNA (Non-inhibitors; Non-responders in our previous publications)^5,6^. Importantly, the transitory inhibition of MSNA in Inhibitors is related to a lack of the stimulus-induced BP increase registered in Non-inhibitors^6^.

Although the MSNA response profile to sudden sensory stimuli has been shown to be an individually reproducible, relatively stable characteristic^6^, it is not shared between monozygotic twins^11^. Hence, it is more likely to be shaped by environmental influence than by genetic sequence. In this respect, it differs significantly from MSNA at rest^11,12^. Changes in MSNA during cognitive stress (3 min of forced mental arithmetic) are correlated to MSNA response profiles following alerting stimuli. Inhibitors showed relatively more inhibition than Non-inhibitors during cognitive stress, suggesting that the underlying neural mechanisms are similar or coupled. It should be noted that Inhibitors and Non-inhibitors respond with a similar MSNA increase during a cold pressor test (performed in the same experimental session as the mental stress test^13^). It thus seems that the MSNA responses that differ between Inhibitors and Non-inhibitors are specific to certain types of stress, rather than being a generalized difference in MSNA responsiveness to all forms of input. Interestingly, phobic syncope patients display exaggerated MSNA inhibition following sudden sensory stimuli, as compared to non-phobic syncope patients (who did not differ from age-matched healthy controls), giving further support to the notion that cortical processing may be involved in shaping sudden stimulus induced MSNA response profiles and associated BP responses^14^.

Taken together, the above findings suggest that the specific sympathetic response profiles i.e., being an MSNA Inhibitor or Non-inhibitor, reveal important information regarding an individual’s short-term BP trend triggered by an environmental stressor. The high frequency with which such stressors are encountered in a modern urban society suggests that these response profiles may have important implications in terms of BP variability and thus long-term BP trends. However, such implications are challenging to explore today because a non-invasive surrogate variable for sudden stimulus induced MSNA inhibition has not been established. An individual’s status as an MSNA Inhibitor or Non-inhibitor and the BP trends coupled to these groups has only been demonstrated with microneurography, which is an invasive, delicate, and time-consuming method only utilized in a handful of labs worldwide. However, the links reported between MSNA inhibition and phobia^14^ as well as cognitive stress^13^ suggest that transient MSNA inhibition is likely to be coupled to cortical processing. Hence, neuroimaging studies could identify cortical signatures that are more clinically accessible as surrogates for microneurography-based MSNA response profiles.

While little is known regarding the role of the brain in relation to MSNA inhibition induced by sudden/stressful stimuli per se, several studies implicate parts of the central autonomic network as likely to be of importance. Goswami et al., for example, report that activation of large-diameter muscle afferents elicit a modest attenuation of MSNA during baroreceptor unloading. They associate this attenuation with reduced activation of insular and anterior cingulate (ACC) cortices, suggesting an integration of somatosensory and baroreceptor afferent input to these regions^15^, which are well established parts of the cortical autonomic network^16^. The rostral ACC/medial prefrontal cortex (mPFC) and insula regions are furthermore implicated in baroreflex control and cardiac arousal^17^. Electroencephalography (EEG) has also been used to reveal a relationship between solitary vasoconstrictor bursts and EEG K-complexes occurring during sleep stage II^18,19^. Finally, while cortical correlates to *skin* sympathetic activation during arousal, gauged by electrodermal responses, have been investigated in man^20,21^, such correlates to the transient response of *muscle* sympathetic nerve activity to sudden stimuli remain relatively unexplored. Because MagnetoEncephaloGraphy (MEG) provides sampling of cortical activations with high temporal resolution and the ability to identify the brain regions generating them with confidence^22^, it offers the possibility to bridge the results above. Our aim herein was to enable a broader range of studies of sudden stimulus/stress-induced MSNA defense mechanisms in relation to cardiovascular reactivity by searching for brain structural and functional correlates to MSNA response profiles.

Using magnetic resonance imaging (MRI) and MEG, we test the hypothesis that cortical thickness and responses within the cortical autonomic network to sudden stressful stimuli correlate with microneurography-based MSNA response profiles. Because the stimuli were sensory, the sensorimotor cortex (Rolandic region) was also included as a pre-selected area of interest in these tests.

## Methods

### Subjects

We recruited participants via public notice boards at the university and invited and included the first 20 volunteers that applied (aged 19 to 45, mean age 31 years, SD 7.7 years; Table 1). The sample size was determined based on previous microneurographic experience^6^. Inclusion criteria were male sex and 18 + years of age. Exclusion criteria were set as any current medical diagnoses or use of medical prescriptions. Informed consent was obtained from all participants. The study was approved by the regional Human Ethics Committee in Gothenburg (Etikprövningsnämnden i Göteborg, dnr 488-12) and conformed to the Declaration of Helsinki. Table 1 presents a summary of these and other subject characteristics that we gathered.

**Table 1:** Subject characteristics.

Group	Non-inhibitor (n = 10)				Inhibitor (n = 10)				All (n = 20)		*p*
Measure	Mean	Std.	Min	Max	Mean	Std.	Min	Max	Mean	Std.	
Age	29.0	6.8	21	41	32.7	8.6	19	45	30.9	7.8	0.30
BMI	24.6	3.7	19.8	34.1	23.7	4.1	19.4	34.4	24.1	3.8	0.58
SBP	120.4	9.8	105	135	115.0	7.6	103	128	117.7	9.0	0.19
MAP	84.2	5.8	73	92	80.0	6.1	67	88	82.1	6.2	0.13
DBP	64.8	7.1	51	73	64.5	8.8	43	74	64.7	7.8	0.93
HR	58.2	9.2	49	82	53.8	5.7	45	62	56.0	7.8	0.22
BI	40.7	12.5	12.5	56.0	47.8	14.6	24.4	70.3	44.3	13.7	0.26
BF	24.6	6.6	12.0	35.3	25.5	5.7	17.6	34.6	25.0	6.1	0.76

Participants grouped by MSNA inhibition. *Std*. standard deviation of the mean, *Min* minimum value, *Max* maximum value, *p p*-value from independent samples T-test for difference between Non-inhibitor and Inhibitor groups, *BMI* body mass index, *SBP* resting systolic blood pressure, *MAP* resting mean arterial pressure, *DBP* resting diastolic blood pressure, *HR* resting heart rate, *BI* resting MSNA burst incidence (per 100 heart beats), *BF* resting MSNA burst frequency (per minute).

### Experimental design

Each subject underwent three separate occasions of controlled experimental paradigms. Microneurography was first used to establish each subject’s MSNA response profile and related BP reactivity i.e., by recording MSNA and BP responses to sudden/stressful stimuli in line with previous works^6,11^. In summary, the consecutively-recruited subject group happened to be evenly split between Inhibitors (n = 10) and Non-inhibitors (n = 10) and, as previously demonstrated, only Non-inhibitors displayed elevated (mean arterial) BP in response to sudden stimuli. Having established each subject’s MSNA inhibition profile, we could then start our search for cortical correlates. MRIs of each subject’s head were used to extract cortical thicknesses (as an index of neural activity) in the central autonomic network regions implicated in MSNA inhibition i.e., the insular cortex and ACC^15–17^. We also generated subject-specific electrophysiological head models for MEG source reconstruction from these individual MRIs. Finally, we recorded MEG while subjects were exposed to sudden/stressful stimuli in line with that which was used to establish their MSNA response profile with microneurography. As with the structural analysis, we limited our search in the MEG source space to the ACC and insular cortex, but also included the Rolandic area. We aimed to perform MSNA and MEG experiments within a 12 month period, and for 19 subjects the median interval was 38 days between studies (range 2–322 days). One outlier, not available for repeated microneurography, had participated in several previous MSNA studies, the shortest interval to the MEG study being 2487 days.

### Stimulation protocols and establishing MSNA response profiles

MSNA was recorded from the peroneal nerve of the left leg. Stressful stimulation was delivered to the left index finger as a train of five transient electric shocks^6^, delivered during five consecutive cardiac intervals, repeated for 72 trials, and is detailed further below. The first shock in each train causes an arousal reaction with the potential to modulate MSNA. Total MSNA depends on burst frequency and burst amplitudes^23^. Averaged stimulus-induced MSNA inhibition is a mixed measure that accounts for both mechanisms, as absent bursts are assigned a zero amplitude. Stimulus-induced MSNA inhibition was defined as previously described^11^. In essence, it is the average post-stimulus amplitude compared to a control period of 8 cardiac intervals immediately preceding each stimulation. It can empirically range from highly negative (e.g., − 200%, corresponding to MSNA bursts after sudden stimuli being 2 × higher in magnitude than those during baseline) to + 100% (i.e. the post-stimulus burst being completely absent—as MSNA bursts are rectified/integrated numbers of efferent nerve action potentials, their magnitude is always non-negative and thus MSNA inhibition cannot exceed 100%). Individuals with MSNA inhibition of more than 30% were defined as Inhibitors^11^. The 30% threshold was selected by quantifying the normal variability during dummy stimuli (i.e., R-wave timed triggers in the absence of stimulation). We have previously determined that for 95% of individuals, the averaged amplitude deviation in relation to dummy stimuli is within ± 30%^11^; a reduction in average burst amplitude of more than 30% was therefore considered an active inhibitory response. Out of 20 consecutively recruited participants, 10 individuals were Inhibitors and 10 were Non-inhibitors. In our experience, the percentage of Inhibitors typically ranges from 50 to 75%^11,13^. The MSNA inhibition values are normally distributed, but with the occasional outlier.

In the present study, we had an outlier whose MSNA inhibition was − 132%. This individual also displayed low baseline MSNA, well within the normal range but making relative change more difficult to determine reliably. Although his absolute MSNA inhibition value may be exaggerated, it was nevertheless assessed (via viewing the raw data) that this subject was displaying sympathetic excitation (and thus non-inhibition per definition). We found no compelling reason to exclude this subject from the study. Furthermore, we make use of non-parametric statistics that are robust against solitary outliers when comparing against our regions of interest (ROI).

The cortical responses to arousal stimuli were examined in a separate session. Sudden stressful electric stimuli were presented during the MEG session in a fashion very similar to that which was used for establishing MSNA response profiles, c.f. above and^6,11^. Briefly, they consisted of constant-current square wave pulses (200 µs duration) applied across a pair of surface ring electrodes positioned on the middle and proximal phalanx of the index finger of the left hand. The strength of the pulses was adjusted prior to the recording for each subject, according to his rating on a Visual Analogue Scale that ranged from 0 (no pain) to 10 (intolerable pain). We aimed for a 7–8 on this scale such that repeated stimuli would be perceived as bearable, but strong enough to continue to elicit transient arousal throughout the whole stimulation period. A single trial consisted of 3 electric pulses, each of which were triggered to arrive 200 ms after the R-wave of the subject’s electrocardiogram (ECG). This specific timing of the stimulus with respect to the R-wave was optimized for evoking MSNA inhibition^5^. The 3 electric pulses within a trial were applied every other heartbeat, instead of 5 consecutive ones used during microneurography, in order to allow a sufficiently long time window between pulses within a trial, without the interference of further stimulation (around 1.5–2 s, depending on the heart rate of the individual subject) to analyze cortical dynamics throughout. Each participant received 72 stimulus trials with an inter trial interval of 30, 45, or 60 s. This interval was varied according to a randomly generated sequence, which was kept the same for all participants in each session. The randomization of the intervals was designed to minimize habituation and expectation effects with respect to the first pulse in each train. Subsequent pulses within a trial were expected by the subjects (they were instructed about the 3-pulse trials) and were meant to enable contrast between sudden/unexpected stimuli and predictable ones.

### Verifying related BP changes

**Figure 1:**
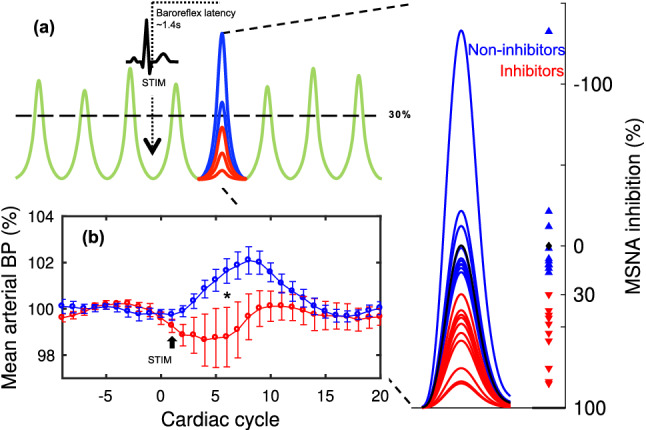
Microneurography and blood pressure recordings. (**a**) Schematic illustration of averaged MSNA inhibition in response to a series of arousal stimuli. The right inset presents the distribution of mean post-stimulus MSNA burst amplitudes observed in each study participant, presented as both synthetic mean bursts and a dot plot. Inhibitors are defined as those displaying an average post-stimulus reduction in mean burst amplitude (MSNA inhibition) of 30% or more following the first (unexpected) stimulus in each train (red). **(b)** The average mean arterial BP (lower left) is presented as a function of cardiac cycle number relative to stimulus presentation in Inhibitors (red, n = 7) and Non-inhibitors (blue, n = 8), error bars represent standard error (SEM). The stimulus train begins at cardiac cycle ‘1’, indicated by the black arrow. BP in response to arousing stimuli in Non-inhibitors is significantly higher as compared to Inhibitors during cardiac interval 6 (*p = 0.021).

To enable confirmation of previous findings of BP differences based on MSNA inhibition, we compared arterial BP reactions between Inhibitors and Non-inhibitors. Arterial BP was monitored continuously using a volume-clamp method with a cuff around the middle phalanx on the third finger (Finometer model 1; cuff size medium, Finapres Medical Systems, Arnhem, the Netherlands) on the same side as the microneurography recording (left side). Normotension was confirmed through the mean value of three consecutive readings in the supine position, at the end of the experiment, by a sphygmomanometer (Omega 1400, cuff size Adult 11; Invivo Research Inc., FL) on the left upper arm. Because 5 participants were recruited from other ongoing studies on MSNA inhibition, with a modified stimulation protocol compared to Donadio et al.^6^, they were excluded from BP analysis. For the remaining 15 subjects, we used Wilcoxon rank sum and Spearman correlations on post-stimulus cardiac interval number 6 because of the expected latency between the presence/absence of a muscle sympathetic burst and the maximal BP effect^24^. The BP changes induced by sudden stimuli for Inhibitors and Non-inhibitors are shown in Fig. 1 and correspond well with previous research^6^. A between-group comparison showed that the mean arterial BP response in Non-inhibitors (n = 8) was significantly elevated as compared to Inhibitors (n = 7, Wilcoxon rank sum; *p* = 0.021). This difference reflected an elevated diastolic pressure (*p* = 0.029) in Non-inhibitors. Comparing individual MSNA inhibition and BP responses did not yield a statistically significant correlation (Spearman, r_s_ = − 0.52, *p* = 0.051), nor did any other variable tested correlate with MSNA inhibition (heart rate, respiration, as well as pulse and systolic BP levels, c.f. below and Table 1).

Wilcoxon rank sum was also used to test for beat-by-beat differences in change of heart rate and pulse pressure between Inhibitors and non-Inhibitors for all post-stimulus cardiac intervals containing a visible difference in mean BP i.e., beats no. 1–15. All *p*-values for tests on heart rate and pulse pressure were non-significant; correction for multiple comparisons was thus not necessary.

Finally, respiratory movements were monitored with a strain-gauge attached to a belt around the lower part of the chest. No systematic effect on respiration was elicited by the electrical stimuli (triggered averaging) nor was MSNA inhibition affected by whether the stimulation coincided with inspiration or expiration.

### MRI image acquisition

T1-weighted (isotropic 1 mm^3^ voxel scan resolution) structural MR images were acquired from all participants. Scans were performed on a Philips Gyroscan 3 T Achieva (Philips, Eindhoven, the Netherlands). The two-channel parallel transmit mode was used for improved signal homogeneity over the field of view and the 32 channel SENSE head coil (same manufacturer as the scanner) was used for receiving the MR signal. Our T1W 3D TFE scan parameters were: flip angle 8°, TE = 3.8 ms, TR = 8.2 ms, SENSE (AP factor 2, RL factor 2.6), TFE factor 120, and 180 sagittal slices.

### Brain morphometry

Grey matter volume is an established measure of structural compliance in relation to functional demands due to changes in synaptic density^25–27^ and has also been coupled to the strength of sympathetic resting activity^28^. We therefore used cortical thickness as a surface-based 1-dimensional index^29,30^ of functional demand. In order to determine cortical thickness, we used a histologically validated method in FreeSurfer^31^. MR T1 images were pre-processed using the fully automated standard reconstruction algorithm provided by the software wherein scans undergo spatial alignment, intensity normalization, skull stripping, white/grey matter segmentation, tessellation, surface smoothing and alignment, atlas labelling, and statistics.

### Regions of interest

Literature-informed ROIs were used in both the structural and functional imaging analysis to improve statistical power and reduce the number of multiple comparisons. We chose two main cortical ROIs (cf *Introduction* and *Limitations*): the insular cortex and rostral ACC (the latter structure corresponds to the ‘ACC’ in the four-region model of the cingulate cortex^32^ and contains the subgenual and pregenual ACC). Together, the insular and anterior cingulate cortices constitute the cortical division of the central autonomic network^16^. Furthermore, the rostral ACC has importance for a great number of evaluative tasks as well as homeostatic regulation^33^. The ROIs were extracted from the Desikan-Killiany atlas in FreeSurfer.

For the cortical thickness estimations, the surface labels *insula* and *rostral ACC* were used. ROI thickness estimates were extracted using the Qdec application in FreeSurfer (v 11.4.2). This method extracts the mean cortical thickness for each ROI in each subject. A bilateral region of interest of rostral ACC was emulated by summing left and right labels and calculating the mean estimates. Statistical analysis was performed in ‘Statistical Package for Social Sciences’ (SPSS, v 22.0). In order to reduce bias from outliers, the non-parametric Spearman correlation was used to test the relationship between regional cortical thickness and stimulus-induced MSNA inhibition.

For the source-level MEG analysis, volumetric versions of the aforementioned atlas regions were extracted with the automatic segmentation volume tool (aseg) in Freesurfer^34^ and used to construct source models based on the individual MRIs. The left and right rostral ACC regions were combined (attempting to distinguish between deep sources in close proximity was deemed unwise) whereas we limited the insula to the side contralateral to stimulation. Because the stimulus we employed was somatosensory, we also included the right central sulcus region (contralateral primary sensory and motor cortices: Rolandic cortex) as an ROI in the MEG analysis.

### MEG acquisition

As is standard for establishing MSNA response profiles, the subjects were instructed to abstain from caffeine, physical exercise, and nicotine for 12 h before the MEG session. Subjects were furthermore instructed to abstain from alcohol for 24 h before the session.

MEG was recorded in a magnetically shielded room (model Ak3B, Vacuumschmelze GmbH) at the NatMEG laboratory (www.natmeg.se) at Karolinska Institutet, Sweden that hosts an Elekta Neuromag® TRIUX system with 102 magnetometers and 204 planar gradiometers. The locations of fiducials and at least 100 additional points from the head surface were digitized with a Polhemus FASTRACK system for coregistration of the MEG data with individual anatomical MRIs. Head position indicator coils monitored the position and orientation of the head throughout the recording. ECG was recorded via 4 electrodes: 2 attached at the collarbones and 2 below the chest close to the waist (positive on right collarbone, reference on left collarbone, ground on the right side of the waist and negative on the left). EOG signals for vertical and horizontal eye movement were recorded to detect and reduce the effects of blink artefacts. An accelerometer was attached to the fingernail of the left index finger to monitor possible finger movements induced by the stimulation to that finger. Participants sat upright in the MEG and were instructed to look at a cross in the center of a projection screen placed in front of them during the recording. The recording time was approximately 60–70 min.

### MEG pre-processing

The MEG datasets were pre-processed with Maxfilter® using temporal signal space separation (tSSS) and head movement compensation with a correlation limit of 0.9 and a 10 s buffer length^35^. The channels with noise were marked manually before applying tSSS. The continuous data was low pass filtered at 40 Hz. The main components of cardiac and ocular artefacts were removed from MEG data using Independent Component analysis (ICA). We typically rejected 2–4 independent components (1–2 Ocular, 1–2 Cardiac) for each subject based on an automated MNE-Python procedure^36,37^ and visual inspection. On average, 60% of the trials had blinks. Avoiding such ocular artefacts during trials was difficult, as eye blink is an expected reaction to unexpected stimuli; however, the number of trials with eye blinks was not correlated with MSNA inhibition (r_s_ = − 0.29, *p* = 0.21). After ICA, the trials were visually inspected and epochs with residual ocular artefacts in the MEG data were rejected from further analysis. We also checked for the effect of finger movement after stimulation. The percentage of trials with finger movement was not correlated with MSNA inhibition (r_s_ = 0.26, *p* = 0.25). On average only 1% of trials included finger movement when excluding two outlier individuals (that had 7% and 38% of trials with finger movement). Excluding these two individuals from the analysis furthermore did not change the reported results; the trials with finger movement were therefore not excluded from the final results. On average, 1.2% of epochs were rejected for each subject.

### MEG source analysis

Time domain source estimates for MEG data were calculated using the linearly constrained minimum variance (LCMV) spatial filtering beamformer method^38^ implemented in the FieldTrip toolbox^39^. Spatial filters were constructed from subject-specific lead fields and data covariance matrices. The lead fields were generated with a realistic single shell model^40^. The data covariance matrix was calculated with all conditions combined together (1.5 s of baseline (the pre-stimulus, ECG R-tag triggered interval before pulse 1)), and 1.5 s intervals after pulses 1, 2, and 3 (which are R-tag triggered in the same way as the pre-stimulus interval) in order to obtain accurate and unbiased estimates of a common spatial filter for all conditions. An interval of +/−5 ms around the time of stimulation was excised from the raw data of all recordings in order to avoid any potential stimulation artefacts. The pre-specified ROIs in each participant’s MRI were discretized to a grid with 5 mm resolution. Spatial filters were then estimated for each grid location and applied to the raw data to obtain source power estimates. The power estimates at each grid position for pulses 1, 2, and 3 were contrasted with the baseline interval. The trial-by-trial time series for each grid point within each ROI was extracted using the spatial filters. Only those vertices/grid locations that were reliably activated within each anatomical ROI (i.e., those whose power exceeded 60% of the peak response within that ROI) were further analyzed. The response from those vertices was then averaged to obtain a single time series for each ROI and for each trial. This time-series was spectrally decomposed in the 5–40 Hz frequency range using a 7-cycle Hanning-tapered sliding window, which was shifted in 5 ms steps. The spectral power of the neural activations after pulses 1, 2, and 3 was normalized with respect to the pre-stimulus interval (i.e., 1.5 s of data time-locked to the heartbeat prior to pulse 1). It is important to note that, up to this point and aside from the ROI selection, the analysis of the MEG data (that resulted in vertices/grid locations for further analysis) did not include any MSNA-inhibition correlation-based search.

Finally, a non-parametric cluster-based permutation test^41^ was used to test whether the power in time–frequency points (for each set of reliably activated vertices/grid locations within each ROI) was significantly correlated with MSNA inhibition (*p* < 0.05, two-sided, 1 000 permutations). The power in the time–frequency points in clusters that exceed the significance threshold was subsequently averaged and used to calculate the correlation with MSNA inhibition. We used the Spearman coefficient for calculating all correlations.

### Statistical analyses

**Figure 2:**
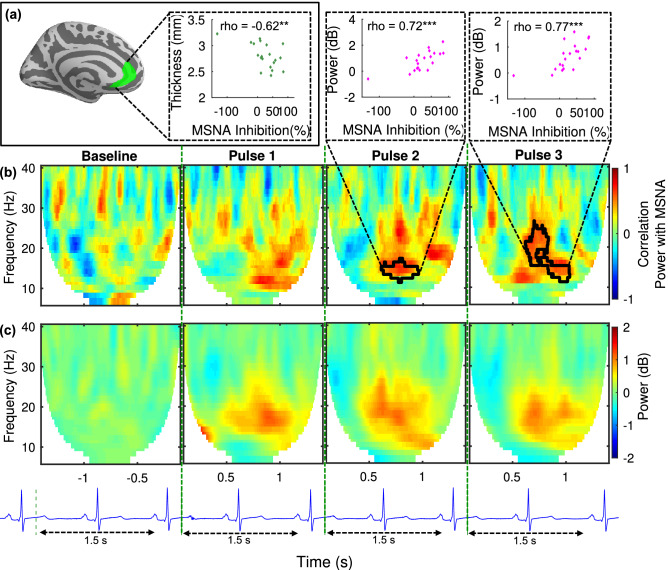
Structural and functional analysis of the anterior cingulate cortex. All power values are relative to the pre-stimulus baseline (n = 20). **(a)** (left) rostral ACC ROI, (right) correlation between cortical thickness and individual MSNA inhibition. **(b)** Correlations between MEG oscillatory power changes and individual MSNA inhibition overlaid with above-threshold clusters (marked with black boundaries). The correlation between average power within the clusters and MSNA inhibition for each subject is presented in the inset above the clusters for pulse 2 and pulse 3 responses. (C) Grand average MEG oscillatory power changes in the 5–40 Hz frequency range in the rostral ACC for each of the three pulses. The schematic ECG shows how stimulation was time-locked to 200 ms after the heartbeat. Spectral power analysis included 1.5 s after each stimulus due to variations in heart rate. **p < 0.01 ***p < 0.001.

**Figure 3:**
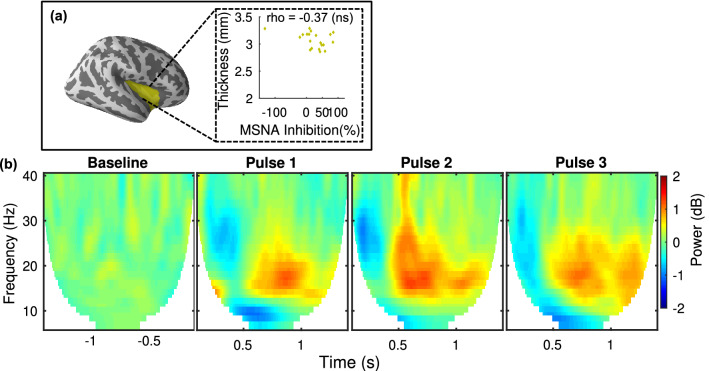
MEG neural oscillatory response and cortical thickness in the Insula. All power is relative to pre-stimulus baseline (n = 20). **(a)** (left) Insula ROI, (right) correlation between cortical thickness and individual MSNA inhibition. **(b)** Grand average MEG oscillatory power changes in the 5–40 Hz frequency range in the Insula for each of the three pulses. No correlations between oscillatory power and MSNA-inhibition were found.

**Figure 4:**
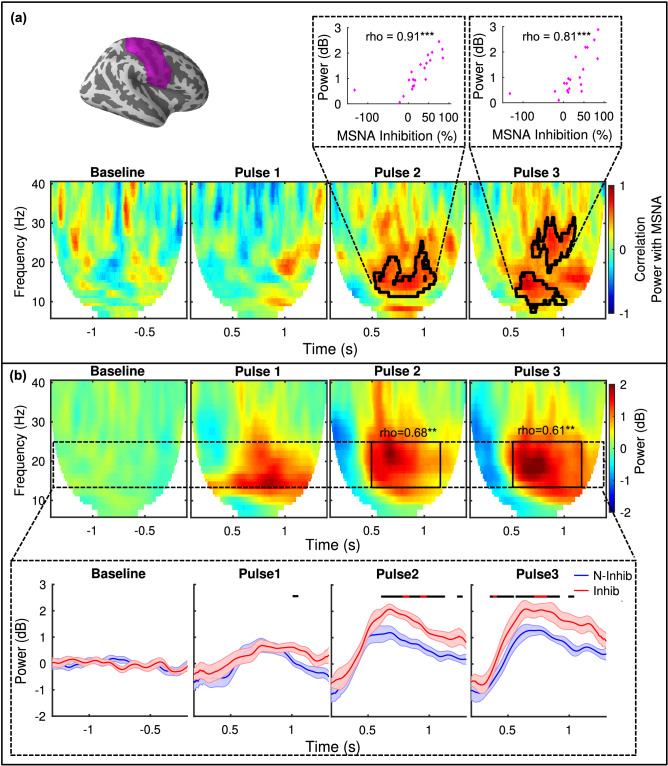
MEG neural oscillatory responses in the Rolandic sensorimotor cortex. All power is relative to pre-stimulus baseline (n = 20). **(a)** (top left) Rolandic ROI. Time–frequency maps of correlations between oscillatory power changes and individual MSNA inhibition values overlaid with above-threshold clusters. Correlations between average power within the clusters and MSNA inhibition is shown above for pulse 2 and pulse 3. **(b)** Grand average oscillatory power changes in the 5–40 Hz frequency range. The responses following all three pulses show early (0 to 0.4 s) beta desynchronization followed by beta rebound (0.5 to 1 + s). A general time–frequency window guided by the clusters in A is shown in black (0.5 to 1.2 s and 13–25 Hz) and average power therein correlates with individual MSNA inhibition (pulse 2: r_s_ = 0.68, *p* = 0.001; pulse 3: r_s_ = 0.61, *p* = 0.005). The average power over time in the beta frequency range (13–25 Hz, dotted windows) is shown below for Inhibitors (red, n = 10) and Non-inhibitors (blue, n = 10), shaded region shows SEM. Bars indicate regions of the time-scale containing a significant group difference (black: *p* < 0.05; red: *p* < 0.01). (**p < 0.01, ***p < 0.001).

Detailed descriptions of statistics regarding MSNA response profile establishment (and related BP differences), MRI cortical thickness and MEG source localization, and response analysis are given in their respective sub-section above. The distribution of MSNA inhibition was assessed by the Shapiro–Wilk test of normality (0.848; *p* < 0.001) and determined to not follow a normal distribution. However, exclusion of the outlier subject (3.3 standard deviations from the mean) provides normally distributed data (0.957; *p* = 0.52). All correlations between ROI data and MSNA inhibition (Figs. 2, 3, 4) were calculated using non-parametric Spearman coefficients, whereas group differences in beat-by-beat BP and heart rate were assessed by Wilcoxon rank sum. Significance bars in Fig. 4 are based on multiple two-tailed t-tests and should only be used for visual guidance. The Bonferroni method was used to correct for multiple comparisons of the pre-selected cortical regions of interest (i.e. insula, rostral ACC, Rolandic).

### Ethics approval statement

Informed consent was obtained from all participants. The study was approved by the regional Human Ethics Committee in Gothenburg (Etikprövningsnämnden i Göteborg, dnr 488-12, add T067-16) and conformed to the Declaration of Helsinki.

## Results

Below we present results from testing the relationship between microneurography-derived MSNA responses *vs* MRI-based measures of cortical thickness and MEG-based measures of cortical responses, respectively.

### ACC thickness correlates negatively with MSNA inhibition

The results of the search for cortical central autonomic network morphology-based correlates to MSNA response profiles are presented in Fig. 2. Two candidate ROIs were pre-selected (see “Introduction” and “Methods” section). We found that cortical thickness in the rostral ACC correlated with individuals’ MSNA responses (n = 20; Spearman, r_s_ = − 0.62, *p* = 0.004). The negative correlation revealed that Non-inhibitors have a larger amount of grey matter in this area. No significant correlation was found in the insular cortex (r_s_ = − 0.37, *p* = 0.10).

### Power in ACC beta correlates positively with MSNA inhibition

The modulations of neural oscillations in the cortical brain regions within the central autonomic network and sensorimotor area that are linked to MSNA response profiles are also presented in Fig. 2. Clusters of time–frequency points with above-threshold correlations were identified through a non-parametric cluster-based permutation test (n = 20; *p* < 0.05). Similar to the structural results, no significant correlation effects between the MSNA arousal response and spectral power changes were found in the insular cortex. In the rostral ACC, however, significant responses were found. Figure 2 includes the average (n = 20) oscillatory power changes in rostral ACC in the 5–40 Hz frequency range that correlate with MSNA for each of the three pulses. The non-parametric permutation test revealed clusters of activity with positive correlations between MSNA inhibition and an increase in power, i.e. synchronization of activity, in the beta (13–25 Hz) band at t ~ 0.75 s post-stimulus in time–frequency space. The correlation was significant in the rostral ACC response to both pulse 2 (r_s_ = 0.73, *p* < 0.001) and pulse 3 (r_s_ = 0.77, *p* < 0.001) but not to pulse 1 (Fig. 2). Finally, a response to stimulation was found in the Insula for which the grand average is similar to that found in rostral ACC, but no correlation with MSNA was detected (Fig. 3).

### Power in Rolandic beta rebound correlates positively with MSNA inhibition

As the stimulus paradigm was somatosensory, we also tested for correlates in the primary sensorimotor cortical responses. The average oscillatory power in the contralateral central sulcus (Rolandic) area shows a typical somatosensory stimulation response with an event-related beta desynchronization followed by resynchronization, i.e. a *beta rebound*^42,43^, in response to all three pulses (Fig. 4). The correlation between spectral power changes and MSNA inhibition for each time–frequency bin is presented in Fig. 4 along with clusters of time–frequency points with above-threshold correlations (n = 20; *p* < 0.05). As was the case with the rostral ACC, MSNA inhibition in these clusters was strongly correlated with beta rebound after pulses 2 (r_s_ = 0.91, *p* < 0.001) and 3 (r_s_ = 0.81, *p* < 0.001) (Fig. 4). The positive clusters indicate that subjects with stronger MSNA inhibition have higher beta rebound power. No above-threshold clusters were found in the response to pulse 1. This might be an amplitude effect, as the power changes after pulse 1 are relatively low (as compared to those after pulses 2 and 3), which could be attributed to a more spatially diffused and non-specific reaction to the unexpected^44,45^.

In order to ascertain whether the Rolandic response could still be correlated with MSNA inhibition using a general search window (i.e., one that could be used as a prior in future studies), we created a more general time–frequency window of 13–25 Hz and 0.5–1.2 s (relative to each pulse) based roughly on the time–frequency cluster (Fig. 4). In this case, the correlations are weaker, but still significant for both pulses 2 (n = 20; r_s_ = 0.68, *p* = 0.001) and 3 (r_s_ = 0.61, *p* = 0.005). In addition to looking at correlations with individual MSNA inhibition, a grouped average beta-band power response for the two subject categories (i.e., Inhibitors and Non-inhibitors) can be used to understand how they differ in terms of the general beta-band power trends as a function of time. Figure 4 includes such an analysis wherein it is evident that the Non-inhibitors have a weaker beta rebound as compared to Inhibitors.

## Discussion

In this study, we searched for cortical measures that correlate with transient MSNA responses to sudden stimuli. To that end, we characterized a group of healthy male individuals with microneurography and used MRI and MEG to examine structural and functional cortical correlates within candidate brain regions that are most likely to be coupled to MSNA inhibition and the sensory stimuli we used, i.e. the central autonomic network and sensorimotor areas. We found significant correlations in the rostral ACC and Rolandic area. The cortical thickness (MRI) of the rostral ACC was negatively correlated with the degree to which individuals inhibited MSNA. In the rostral ACC, MSNA inhibition was found to correlate with the magnitude of stimulus-induced beta synchronization (MEG). The involvement of the rostral ACC is thus implicated by results from two different brain imaging modalities. Insula was also postulated as an ROI. While it displayed a response to stimulation that is similar in nature to that which was observed in the rostral ACC, that response failed to show any significant associations with MSNA inhibition. Finally, we found that the Rolandic beta rebound response measured with MEG correlated highly with MSNA response profiles. As such, we demonstrate statistically significant structural and functional cortical indices of a human sympathetic defense response.

### ACC and defense reactions

Our results reveal new aspects of cortical processing related to transient MSNA defense responses. Sudden stressful stimuli, as a feature of our experimental model, can be viewed as the triggering step in a series of reactions involved in defensive behavior which has been referred to as the defense cascade^46^. Depending on the stimulus type and intensity as well as the situational context, different responses may be elicited, including a fight-or-flight mode of action. The insular cortex and ACC have both been described as part of a cortical network that modulates autonomic functions^47–52^ and may be coupled to arousal-related MSNA reactions (e.g., inhibition). However, the rostral ACC response that correlates with MSNA inhibition does not occur in time until after inhibition has taken place (Fig. 2); it therefore cannot be a direct *modulator* of inhibition. The rostral ACC response is rather *coupled* to MSNA inhibition: it could, for example, be a ‘simple’ reflection of it, the result of the propagation of an unrelated process running in parallel to it, or, as we argue in the following, an indicator of an important connection between (rapid) low-level physiological stress reactions (i.e., MSNA inhibition) and (slower) high-level evaluative processes that are initiated by stress.

Since the MSNA profile reflects an individual’s defense response, its anatomical and functional correlation with the rostral ACC (without similar correlations revealed in the insula) supports the notion that the rostral ACC is involved in evaluation of alerting stimuli and modulation of reactions pertaining to the concept of defense. The structural correlation suggests a longer standing interplay between such stimuli and reactions, whereas the functional correlation might reveal a neural signature of a chosen response strategy (cf Beta rebound below). This brain region has been found to influence behavior via suppression of innate defensive reactions and facilitation of implicit or explicit avoidance reactions^53^. Such a role is relevant with respect to other cognitive domains that are also linked to the ACC. For example, the ACC has importance for a great number of evaluative tasks, including perceived social standing^33,54^, which might be expected to modulate patterns of behavior and responses to social threat. ACC activations in individuals with urban vs. rural upbringing (differing in abundance of sudden sensory stimuli) furthermore differ significantly during stress tasks^55^.

### Rolandic beta rebound and defense reactions

As shown previously^6^, and replicated herein, the brief (within a single heartbeat) MSNA inhibition (or lack thereof) following a sudden/stressful stimulus is associated with BP responses. Intriguingly, there is evidence suggesting that sympathetic activity can be modulated by cells in the sensorimotor cortex. Retrograde tracing in animals has revealed connections between the sensorimotor cortex, including premotor areas, and the nucleus of the solitary tract and rostral ventrolateral medulla^56,57^ as well as the kidney^58^ and adrenal glands^59^. In the two latter studies, the mPFC/ACC was also implied. However, the inhibition of sympathetic bursts is more or less immediate following pulse 1, whereas the ensuing beta rebound in the sensorimotor region occurs a few seconds later, and roughly overlaps with the BP response. The close relationship between Rolandic beta rebound and MSNA inhibition may therefore be a reflection of different branches within the defense cascade with very different time-scales, as we detail below.

Cortical desynchronization followed by resynchronization in the beta-band, i.e. beta rebound, is typical of somatosensory stimulation in general^42,43^. In the motor cortex, it is observed for voluntary and imagined movements and in the somatosensory cortex, e.g., for tactile and electric stimulation^43^. It has been suggested to represent ‘idling of the cortex’^60^, cortical deactivation^61^, active inhibition of the cortex by somatosensory afferents^62^, and somatosensory gating^63,64^, while also being related to the concentration of GABA, a neural inhibitor^64,65^. A broader review of beta oscillations suggests that it is an indicator for maintaining status quo^66^.

Based on our results and what is known about the beta rebound phenomenon in general, we theorize that after being presented with a sudden stimulus (pulse 1) that initiates a defense reaction, the increase in beta rebound following pulses 2 and 3 (Fig. 4) is likely to reflect gating/filtering of new information via active inhibition or deactivation of the cortex. Such a neural effect could serve to maintain the status quo of the newly-initiated defense reaction. The correlation between the Rolandic beta rebound power and the initial MSNA inhibition could be indicative of a joint reaction, i.e. a wider response pattern, adopted by Inhibitors vs. Non-inhibitors. In essence, stronger filtering of additional sensory inputs after a sudden stimulus may be a cortical *reflection* of a response strategy accompanying increased inhibition of vasoconstrictor activity. The latter is what generates an increase in blood flow to skeletal muscles (supporting maintenance of a full-fledged fight-or-flight response) and, aside from improved performance during physical exertion, blunts blood pressure responses. Non-inhibitors can then be thought of as more receptive to further processing of additional stimuli and/or not fully inclined towards fight or flight. This is then associated with retained vasoconstrictor activity. The consequences for Non-inhibitors are stimulus-induced BP transients^6^ (Fig. 1).

### Future perspectives

Future work should be directed towards detailed cortical circuit-mapping of areas involved in this MSNA defense response, long-term effects on circulatory homeostasis^67^, and identification of potential response-modulating factors. In light of the fact that BP fluctuates during sleep, and sleep stage 2-related EEG K-complexes are associated with blunted baroreflex control of MSNA and heart rate^18,68^, studies on stimulus-induced beta rebound, MSNA inhibition, and homeostasis should also consider the sleep-waking cycle. Regarding MSNA response modulation, training with EEG-based feedback on beta reactivity has already been used for Parkinson’s disease^69^ and would be interesting to study in the context of this paradigm. An open question is whether such an intervention targeting beta modulation would have any impact on MSNA response profiles. Perhaps more importantly, it remains to be seen if Non-inhibitors can alter their position in the inhibition spectrum, reduce the short-term BP increase triggered by environmental stressors, and thus improve their long-term cardiovascular health.

### Limitations

There are some limitations to this study that point to follow-up studies of interest. While possible, the prospect of carrying out microneurography and MEG in parallel in the context of a more all-inclusive session is extremely challenging. The correlations put forth are indeed limited by the fact that the establishment of MSNA response profiles and the MEG recordings were done on separate occasions. However, MSNA response profiles have been shown to be stable^6^, and insights into cortical activity related to the inhibition response rest on correlations that are strong (r_s_ = 0.91, *p* < 0.001 for MEG-detected Rolandic beta), despite the separate recording occasions and the modest sample size. Analyses were furthermore mainly limited to a few cortical regions associated with autonomic functions (i.e., the rostral ACC and insula) and sensorimotor processing (i.e., the Rolandic area); it is therefore possible that other cortical responses may be of relevance. Further full-brain type analyses may thus generate results of interest, both in terms of sympathetic inhibition, as well as the various brain areas involved in the processing of alerting stimuli. However, such analysis would require a large sample size in order to meet significance thresholds.

While our ROIs might seem spatially blunt in comparison with fMRI clusters, further subdivision of our ROIs was deemed inappropriate for statistical reasons. Given the varying locations of cortical activations related to sympathetic activity in previous studies, we could not aim to be more specific without increasing the risk of missing important sources of activation in this inaugural study.

Furthermore, the time–frequency-analysis reveals patterns of synchronization/desynchronization on a highly resolved time-scale. However, it does not directly separate between other common neurophysiological measures, such as excitation/inhibition or increased/decreased metabolic activity. Finally, results are limited by all participants being male; further studies on females are warranted^70^.

## Conclusions

This study identifies cerebral cortex areas linked to peripheral MSNA response profiles elicited by sudden/stressful stimuli. These include strong correlations in parts of a recognized network for autonomic activity, but surprisingly also in sensorimotor areas. We interpret these findings as a novel link between autonomic function and sensory/motor processing and suggest this is evidence of a joint pattern elicited in defense reactions such as fight-or-flight. Differences in stimulus-induced Rolandic beta rebound, as detected with MEG, indicate a possible clinically accessible surrogate for MSNA response profiles. Given the fact that the Rolandic area is well sampled also by EEG, the development of clinical prognostic indicators based on this sudden stimulus paradigm during EEG recording could enable more widespread use in research on short- and long-term consequences of defense reactions.

## Data Availability

The data supporting the findings of this study are available from the corresponding author upon reasonable request.
